# Effect of bone loss on the fracture resistance of narrow dental implants after implantoplasty. An *in vitro* study

**DOI:** 10.4317/medoral.24624

**Published:** 2021-06-20

**Authors:** Bruno Leitão-Almeida, Octavi Camps-Font, André Correia, Javier Mir-Mari, Rui Figueiredo, Eduard Valmaseda-Castellón

**Affiliations:** 1 DDS, MS. Universidade Católica Portuguesa, Faculty of Dental Medicine, Center for Interdisciplinary Research in Health (CIIS), Viseu, Portugal; 2 DDS, MS. Oral Surgery and Implantology, Faculty of Medicine and Health Sciences, University of Barcelona, Barcelona, Spain; 3 DDS, PhD. Universidade Católica Portuguesa, Faculty of Dental Medicine, Center for Interdisciplinary Research in Health (CIIS), Viseu, Portugal; 4 DDS, MS, PhD. Oral Surgery and Implantology, Faculty of Medicine and Health Sciences, University of Barcelona, Barcelona, Spain

## Abstract

**Background:**

Implantoplasty (IP) involves polishing of the exposed surface of implants affected by peri-implantitis (PI). A study was made to determine whether the degree of bone loss influences the fracture resistance of implants with or without IP.

**Material and Methods:**

An *in vitro* study was carried out on 32 narrow (3.5 mm) dental implants with a rough surface and external hexagonal connection. Implantoplasty was performed in half of the implants of the sample. Both the IP and control implants were divided into two subgroups according to the amount of bone loss (3 mm or 7.5 mm). Standardized radiographic assessment of implant width was performed using specific software. The main outcome variable was the maximum compression force (Fmax) of implants when subjected to static resistance to fracture tests. Implant fractures were subsequently analyzed by scanning electron microscopy. A descriptive and bivariate analysis of the data was performed.

**Results:**

Significant changes in implant width were observed after IP (*p*<0.05). No significant differences between IP and control implants were recorded in terms of the Fmax values in the two bone loss subgroups (3 mm: control 854.37N 195.08 vs. IP 752.12N 186.13; *p*=0.302, and 7.5 mm: control 548.82N 80.02 vs. IP 593.69N 111.07; *p*=0.370). Greater bone loss was associated to a decrease in Fmax, which proved significant for the control implants (*p*=0.001). Fractures were more frequently located in the platform (n=13).

**Conclusions:**

Implants with more apical bone levels appear to be more susceptible to fracture. On the other hand, IP does not seem to significantly decrease the fracture resistance of narrow (3.5 mm) platform dental implants with external hexagonal connections. The fact that most fractures occur in the platform area indicates that the latter is exposed to more mechanical stress.

** Key words:**Peri-implantitis, dental implants, compressive strength, titanium, implantoplasty.

## Introduction

Peri-implantitis (PI) is a common disease that affects an important number of patients with dental implants (1,2). This complication leads to progressive peri-implant bone loss, creating defects of different anatomical characteristics, shapes and sizes (3).

Different approaches have been suggested for the treatment of PI, ranging from non-surgical to surgical options. Although a number of authors have described different resective and/or regenerative protocols, some controversy remains regarding the most effective treatment for PI (4-6). Non-surgical therapies seem to be mostly ineffective in preventing disease progression in the presence of moderate or severe PI, though some reports claim otherwise (7). On the other hand, surgical techniques are usually considered to be more predicTable, since they seem to hinder the progression of bone loss (8,9).

Implantoplasty (IP) involves polishing of the exposed rough surface of implants presenting bone loss, with the purpose of detoxifying and smoothening these areas to prevent biofilm accumulation (6,10). However, a number of concerns have been raised, such as bone necrosis due to increased temperature, local and systemic toxicity of titanium particles released during IP, and a reduction of resistance to fracture (11,12). It is therefore important to determine whether IP is a safe technique that does not compromise the long-term prognosis of dental implants. Several *in vitro* reports seem to indicate that IP does not significantly reduce the mechanical resistance of dental implants (13,14). However, a number of other variables may also affect this parameter. For example, the amount of bone loss resulting from PI inevitably modifies the mechanical equilibrium of the implant-abutment-restoration complex, and can lead to complications related with the prosthetic components and implants (15). Indeed, bone loss together with other factors such as implant diameter, crown-to-implant ratio (CIR) and bruxism have been associated with an increased risk of dental implant fractures (16). The implant design and connection might also be important in relation to mechanical resistance (17,18). As mentioned, implants with bone loss often require IP. This procedure reduces the thickness of the implant walls, which in turn can weaken the implant (19). Based on finite element analysis, IP has been associated to a 10% decrease in implant resistance to fracture, independently of the bone level. Also, it is important to underscore that a critical threshold might be reached when more than half of the length of the implant has lost bone support (20).

Due to the scarcity of scientific data for supporting clinical decisions, an *in vitro* study was carried out to analyze the influence of bone loss upon the fracture resistance of narrow dental implants with hexagonal external connections with and without IP.

## Material and Methods

**Figure 1:**
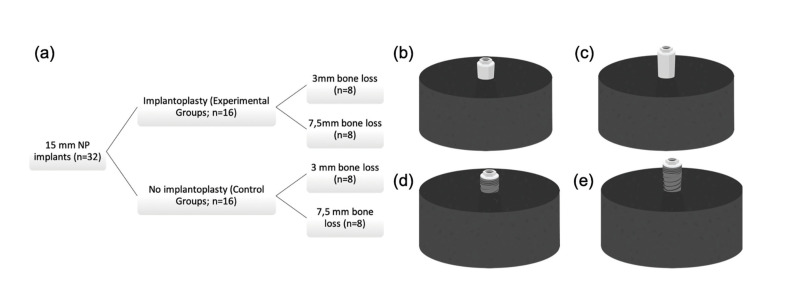
(a) Study design, groups and sub-groups; (b) 3 mm IP sample; (c) 7.5 mm IP sample; (d) 3 mm control sample; (e) 7.5 mm control sample. NP: narrow platform.

An experimental *in vitro* study was made of 32 titanium-type V narrow platform (3.5 mm) dental implants measuring 15 mm in length, with a rough surface and a hexagonal external connection (Ocean E.C., Avinent Implant System S.L., Santpedor, Spain). Sixteen implants were randomly established as control group, while the remaining 16 served as the IP group. In turn, two additional subgroups of 8 implants each were established according to the amount of simulated bone loss (3 mm or 7.5 mm) (Fig. 1).

All implants were embedded in standardized bone-like resin casts (EA 3471 A and B Loctite®, Henkel AG & Company, Düsseldorf, Germany) with ≥ 3 GPa modulus of elasticity according to the International Organization for Standardization (ISO) 14801:2016 (third edition) (Fig. 1), and received a customized hemispherical loading abutment. The protocol used is similar to that described in a recent paper (14).

- Implantoplasty

Implantoplasty was performed according to the technique described by Costa-Berenguer *et al*. (13). In short, an oval-shaped tungsten carbide bur (H379 314 023; Komet Dental, Lemgo, Germany) and two silicon carbide polishers (9618 314 030 and 9608 314 030; Komet Dental, Lemgo, Germany) were used to remove and polish all the exposed areas of each implant with a high-speed handpiece. The procedure was performed by an experienced surgeon (BLA) with 2.8x magnification loupes (Galilean HD and Focus™ LED 6000k, ExamVision ApS, Samsø, Denmark).

- Radiographic assessment of implant width

Modifications of implant width were evaluated radiographically according to the procedure described by Camps-Font *et al*. (21) using plain X-rays and then rotating them 120º and 240º using standardized mounts. All measurements were performed with ImageJ v.1.51 (National Institutes of Health, Bethesda, MD, USA) by a calibrated investigator (BLA) under 400x amplification. Six random implants were assessed twice to test intra-examiner agreement and consistency. The intraclass correlation coefficients (ICCs) were 0.96 (95% confidence interval (95%CI) 0.93 to 0.98; *p*<0.001) and 0.96 (95%CI 0.92 to 0.98; *p*<0.001).

Three reference areas were selected for the measurements: length at the middle of the first thread (R1), tenth thread (R2) and at the end of the prosthetic screw hole (R3), perpendicular to the long axis of the implant. Reference point R3 could not be assessed in the 3 mm subgroup, because this area was embedded in radiopaque resin. The mean measurements of the IP group were subtracted from their control analogues, thus obtaining mean reduction of the implant at each reference point.

- Fracture tests

Resistance to fracture tests were performed in each group to determine the maximum compression force (Fmax) reached before implant fracture occurred (main outcome variable). This procedure was similar to that described by Leitão-Almeida *et al*. (14), except for the amount of implant inserted in the resin and the length of the load abutment. In brief, 7.5 mm-high metal hemispheric load abutments (n=32) were placed on each implant using prosthetic screws (Avinent® Implant System, Santpedor, Spain) at 32 N/cm. All tests were performed in accordance with the UNE-EN ISO 14801:2016 (third edition) guideline parameters, except for supracrestal exposure of the 7.5 mm subgroup. A universal mechanical testing machine (MTS Bionix 370 Load Frame; MTS®, Eden Prairie, USA) applied compression force to the implants with an MTS Load Cell 661.19H-03 of 15 kN capacity. Compression forces were applied at a constant angle of 30 degrees from the vertical axis. Tests were controlled using MTS Flextest 40 (MTS®, Eden Prairie, USA) that recorded real-time data and measured Fmax.

A descriptive analysis of the fractured implants was made from photographs taken with a scanning electron microscope (SEM) (Quanta 200, FEI, Hilsboro, OR, United States).

- Statistical analysis

Previous results from Gherke *et al*. (17) were used to perform the sample size calculation using Stata v.14 (StataCorp, College Station, USA). Considering Fmax as the primary outcome measure, an analysis of variance (ANOVA) with an risk of 0.05 and a statistical power of 80% was performed. Assuming a standard deviation of 500 N, the sample size was established as 8 implants per group.

Scale variables (Fmax and implant width) were explored with the Shapiro-Wilk test, P-P scatter plots and box plots. The interquartile range (IQR) and median were reported when normal data distribution was rejected. The mean and standard deviation (SD) were employed in the presence of a normal distribution.

To analyze the effect of the group (IP or control) and subgroup (bone loss of 3 mm or 7.5 mm) upon Fmax, and the interaction between these two variables, two-way ANOVA was performed. The ANOVA assumptions were assessed using the Shapiro-Wilk test for normality and Levene’s test for homoscedasticity. Pairwise comparisons were made using Tukey’s correction for multiplicity of contrasts. An unpaired t-test was used to identify differences in implant width between control and IP implants. In each area of interest, Pearson correlation coefficients were computed to quantify the correlation between implant width and Fmax. Pearson’s 2 test or Fisher’s exact test were performed for categorical variables.

The statistical analysis was carried out with Stata14 (StataCorp®, College Station, TX, USA). The level of statistical significance was set at *p* < 0.05.

## Results

- Fracture tests

**Figure 2:**
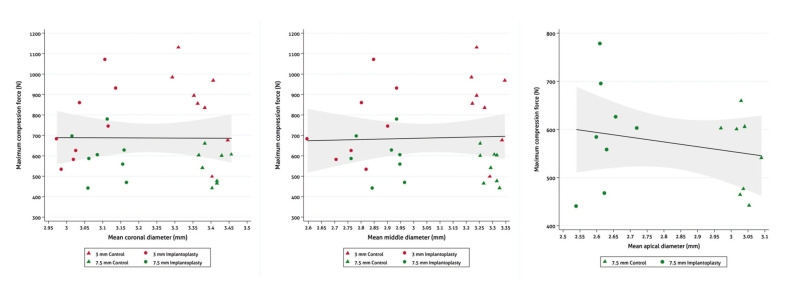
Scatter plot assessing the relationship between maximum compression force (Fmax) and mean sample diameter.

**Figure 3:**
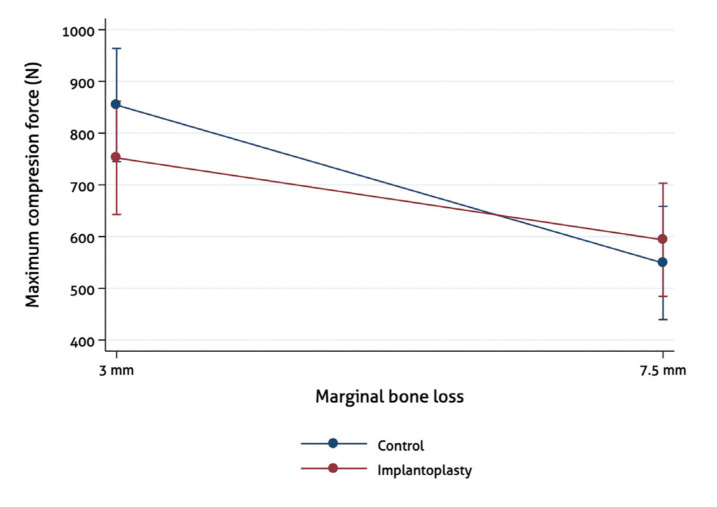
Relationship between maximum compression force (Fmax) and the amount of bone loss.

No correlations were observed between implant wall width and Fmax at any of the reference points (Fig. 2). There was no significant decrease in Fmax when comparing control and IP samples within the same bone loss subgroup (3 mm: control 854.37N 195.08, IP 752.12N 186.13, *p*=0.302; 7.5 mm: control 548.82N 80.02, IP 593.69N 111.07, *p*=0.370) (Table 1, Fig. 3).

**Table 1:**
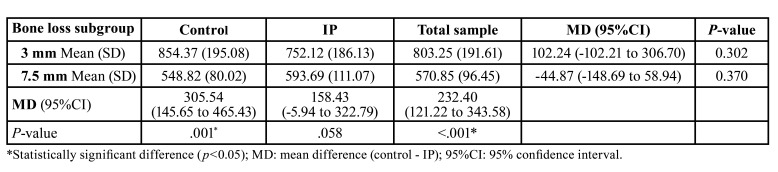
Mean fracture resistance (N) of the bone loss subgroups in IP and control implants.

A significant decrease in Fmax was observed in the 7.5 mm bone loss subgroup in the control samples (mean difference (MD) 305.54N 145.65-465.43, *p*=0.001), and the effect of IP was similar in each bone loss subgroup (Table 1, Fig. 3).

**Figure 4:**
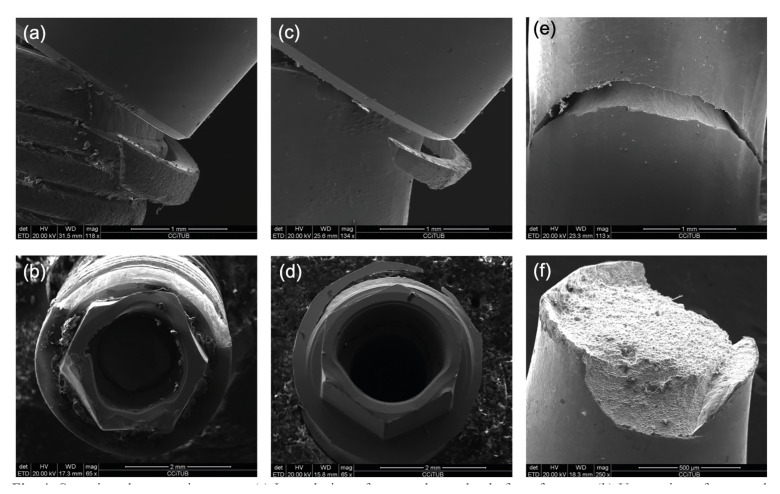
Scanning electron microscopy. (a) Lateral view of a control sample platform fracture; (b) Upper view of a control sample platform fracture; (c) Lateral view of an IP sample platform fracture; (d) Upper view of an IP sample platform fracture; (e) Detail of implant body fracture in an IP sample; (f) Detail of prosthetic screw fracture.

All control and 13 of the 16 IP implants fractured at platform level (Fig. 4). In the IP group, two implant body (Fig. 4) and one prosthetic screw fractures were also observed (Fig. 4).

- Radiographic assessment of implant width

The mean reductions in implant width after IP are reported in Table 2. Implantoplasty was associated to a statistically significant decrease in width at the observed reference points in all subgroups (*p* ≤ 0.05, independent samples t-tests). The magnitude of the decrease was also similar across the bone level subgroups (*p* > 0.05, one-way ANOVA).

No correlations were observed between implant wall width and Fmax at any of the reference points (Fig. 2). There were no perforations of the inner threads in any of the samples.

**Table 2:**
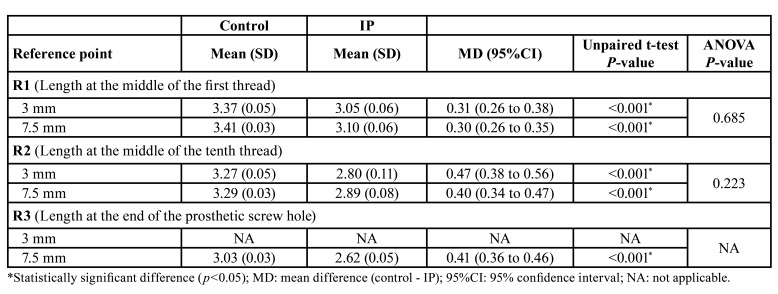
Mean implant width (mm) in the IP and control groups at each reference point (n=32).

## Discussion

Based on the results obtained, IP does not seem to have a significant impact upon the resistance to fracture of narrow platform implants with an external hexagonal connection (Table 1). On the other hand, the amount of bone loss appears to be a relevant factor in relation to fracture resistance, since the Fmax required to fracture implants in the 7.5 mm subgroups was significantly lower than in the 3 mm subgroups (3 mm: 803.25N 191.61; 7.5 mm: 570.85N 96.45; *p*<0.001) (Table 1). Thus, clinicians should be aware that narrow diameter implants with significant bone loss might be more likely to suffer fractures, and that IP does not seem to add any additional risk. Although the mean fracture resistance of IP implants decreased when bone loss increased, this decrease was not statistically significant. A possible explanation for this is that most fractures occurred in the coronal region of the implant (platform area), indicating that this appears to be the most fragile area. Future research should assess whether these results are also valid for internal connection implants.

As expected, a significant reduction in implant width was observed at all reference points due to the IP procedure. Several authors have emphasized that implant diameter affects fatigue behavior of the fixtures, and that IP probably reduces the forces required to reach a critical stress point (15,20,22). The present report appears to contradict this statement, however, since the mean Fmax values of the control versus IP implants were similar. Nevertheless, it is important to stress that IP is not the only variable that should be considered when analyzing the mechanical resistance of dental implants with PI. Indeed, recent studies have shown that implants with internal connections or with an unfavorable CIR seem to be more susceptible to fracture, and that parafunctional habits, implant design and base material can also affect implant strength - thus indicating that these variables also need to be taken into account (13-15,21). Likewise, our results suggest that the amount of bone loss appears to be a more relevant parameter than IP. A reduction of 305.54 N (95%CI 145.65 to 465.43; *p*=0.001) was observed in the control implants when the bone level shifted from 3 mm to 7.5 mm. The IP implants also presented a difference of 158.43 N (95%CI -5.94 to 322.79; *p*=0.058) (Table 1), which is in accordance with previous reports on the impact of bone loss and increasing pocket depths upon dental implant fractures (16).

Using finite element analysis, Tribst *et al*. (20) found that implants with lower insertion levels might increase damage to the bone. Also, IP increases stress in the implant and prosthetic screw, and there is a critical threshold when the inserted part of the implant is smaller than the exposed part. Similar methodology was employed by other authors who also found that the implant embedding depth affects resistance to fracture (23). All these outcomes seem to be confirmed by the present study.

The platform area of narrow implants with hexagonal external connections seems to be more fragile than the body, since all control implants fractured at this point. In the 3 mm bone loss subgroup, all implants (n=16) fractured at platform level. However, in the 7.5 mm bone loss subgroups, two IP implants fractured in the body area and, in one case, the prosthetic screw broke - thus suggesting that IP might reduce mechanical resistance of the implant body with increasing bone loss. Consistent with the present findings, other authors have also reported deformations at the platform border in all tested samples, reinforcing the idea that the platform area might be more susceptible to increased forces (17). When regular platform implants are subjected to IP, body fractures are more common in comparison with those observed in control implants, thus suggesting that IP weakens the implant body (13).

Some important clinical messages might be drawn from the present results. On one hand, clinicians should be aware that deep peri-implant bone defects are a risk factor for implant fracture. On the other hand, even though IP reduces the thickness of the implant walls, it does not seem to decrease the resistance to fracture of the fixtures. One might argue that this study simulates a very adverse clinical situation. Indeed, it is uncommon to find single-unit narrow diameter fixtures with deep peri-implant bone defects in the daily practice. In our opinion, this can also be seen as an advantage since it probably indicates that IP is unlikely to affect the fracture resistance in more favorable scenarios where regular- or large-platform implants are involved. Also, splinted restorations supported by several narrow implants are likely to have a better mechanical behavior and therefore less risk of fracture (24).

The *in vitro* design of the present study implies a number of limitations. First of all, the IP procedures were not fully standardized. However, it is unlikely that this limitation could have affected the results, since the implant width radiographic analysis showed similar reductions for both subgroups. On the other hand, IP was performed while holding the implant with the hand. This fails to reproduce the real-life clinical scenario, where the access can affect the outcome of the technique. Nevertheless, this method has been used previously, so comparisons can be made with the results of other authors (13,14,21). Another possible drawback is related to the fact that static compressive load testing may fail to replicate the complex daily oral function of patients (25). However, this methodology was selected in order to comply with ISO guideline 14801:2016 (third edition). On the other hand, the use of dynamic fatigue tests would increase the external validity of the results, and should be considered in future research. Also, the present report only evaluated 3 mm- and 7.5 mm-high peri-implant horizontal bone defects. Still, these subgroups may be interpreted by clinicians as respectively representing initial or advanced peri-implantitis cases. Finally, different prosthetic materials might have an impact upon the mechanical dynamics of the implants, and additional studies are needed to assess these variables.

Within the limitations of the present *in vitro* study, advanced bone loss should be considered a risk factor when assessing the resistance to fracture of narrow diameter implants with external hexagonal connections. Although IP significantly reduces the thickness of the implant walls, it does not seem to significantly alter the mechanical resistance of dental implants with the abovementioned features.

## References

[1] Mir-Mari J, Mir-Orfila P, Figueiredo R, Valmaseda-Castellón E, Gay-Escoda C (2012). Prevalence of peri-implant diseases. A cross-sectional study based on a private practice environment. J Clin Periodontol.

[2] Kordbacheh Changi K, Finkelstein J, Papapanou PN (2019). Peri-implantitis prevalence, incidence rate, and risk factors: A study of electronic health records at a US dental school. Clin Oral Implants Res.

[3] García-García M, Mir-Mari J, Benic GI, Figueiredo R, Valmaseda-Castellón E (2016). Accuracy of periapical radiography in assessing bone level in implants affected by peri-implantitis: a cross-sectional study. J Clin Periodontol.

[4] Smeets R, Henningsen A, Jung O, Heiland M, Hammächer C, Stein JM (2014). Definition, etiology, prevention and treatment of peri-implantitis-a review. Head Face Med.

[5] Chan H, Lin G, Suarez F, MacEachern M, Wang H (2014). Surgical management of peri-implantitis: a systematic review and meta-analysis of treatment outcomes. J Periodontol.

[6] Schwarz F, John G, Schmucker A, Sahm N, Becker J (2017). Combined surgical therapy of advanced peri‐implantitis evaluating two methods of surface decontamination: a 7-year follow-up observation. J Clin Periodontol.

[7] Estefanía-Fresco R, García-de-la-Fuente AM, Egaña-Fernández-Valderrama A, Bravo M, Aguirre-Zorzano LA (2019). One-year results of a non-surgical treatment protocol for peri-implantitis. A retrospective case series. Clin Oral Implants Res.

[8] Karlsson K, Derks J, Håkansson J, Wennström JL, Petzold M, Berglundh T (2019). Interventions for peri-implantitis and their effects on further bone loss. A retrospective analysis of a registry‐based cohort. J Clin Periodontol.

[9] Esposito M, Grusovin MG, Kakisis I, Coulthard P, Worthington HV (2008). Interventions for replacing missing teeth: Treatment of perimplantitis. Cochrane Database Syst Rev.

[10] Dalago HR, Perrotti V, Torres de Freitas SF, Ferreira CF, Piattelli A, Iaculli F (2019). Prospective longitudinal comparison study of surgical therapies for peri-implantitis: 3-year follow-up. Aust Dent J.

[11] Safioti LM, Kotsakis GA, Pozhitkov AE, Chung WO, Daubert DM (2017). Increased levels of dissolved titanium are associated with peri-implantitis - a cross-sectional study. J Periodontol.

[12] Suárez-López del Amo F, Garaicoa-Pazmiño C, Fretwurst T, Castilho RM, Squarize CH (2018). Dental implants‐associated release of titanium particles: A systematic review. Clin Oral Implants Res.

[13] Costa-Berenguer X, García-García M, Sánchez-Torres A, Sanz-Alonso M, Figueiredo R, Valmaseda-Castellón E (2018). Effect of implantoplasty on fracture resistance and surface roughness of standard diameter dental implants. Clin Oral Implants Res.

[14] Leitão-Almeida B, Camps-Font O, Correia A, Mir-Mari J, Figueiredo R, Valmaseda-Castellón E (2020). Effect of crown to implant ratio and implantoplasty on the fracture resistance of narrow dental implants with marginal bone loss: an in vitro study. BMC Oral Health.

[15] Chan H L, Oh W S, Ong HS, Fu J H, Steigmann M, Sierraalta M (2013). Impact of implantoplasty on strength of the implant-abutment complex. Int J Oral Maxillofac Implants.

[16] Sánchez-Pérez A, Moya-Villaescusa MJ, Jornet-García A, Gomez S (2010). Etiology, risk factors and management of implant fractures. Med Oral Patol Oral Cir Bucal.

[17] Gehrke SA (2015). Importance of crown height ratios in dental implants on the fracture strength of different connection designs: An in vitro study. Clin Implant Dent Relat Res.

[18] Gehrke SA, Souza dos Santos Vianna M, Dedavid BA (2014). Influence of bone insertion level of the implant on the fracture strength of different connection designs: An in vitro study. Clin Oral Investig.

[19] Bertl K, Isidor F, von Steyern PV, Stavropoulos A (2021). Does implantoplasty affect the failure strength of narrow and regular diameter implants? A laboratory study. Clin Oral Investig.

[20] Tribst JPM, Dal Piva AM de O, Shibli JA, Borges ALS, Tango RN (2017). Influence of implantoplasty on stress distribution of exposed implants at different bone insertion levels. Braz Oral Res.

[21] Camps-Font O, González-Barnadas A, Mir-Mari J, Figueiredo R, Gay-Escoda C, Valmaseda-Castellón E (2020). Fracture resistance after implantoplasty in three implant-abutment connection designs. Med Oral Patol Oral Cir Bucal.

[22] Shemtov‐Yona K, Rittel D, Levin L, Machtei EE (2014). Effect of dental implant diameter on fatigue performance. Part I: mechanical behavior. Clin Implant Dent Relat Res.

[23] de la Rosa Castolo G, Perez SVG, Arnoux P J, Badih L, Bonnet F, Behr M (2018). Mechanical strength and fracture point of a dental implant under certification conditions: A numerical approach by finite element analysis. J Prosthet Dent.

[24] Goiato MC, Andreotti AM, Dos Santos DM, Nobrega AS, de Caxias FP, Bannwart LC (2019). Influence of length, diameter and position of the implant in its fracture incidence: A systematic review. J Dent Res Dent Clin Dent Prospects.

[25] Hattori Y, Satoh C, Kunieda T, Endoh R, Hisamatsu H, Watanabe M (2009). Bite forces and their resultants during forceful intercuspal clenching in humans. J Biomech.

